# Improved Cardiovascular Tolerance to Hemorrhage after Oral Resveratrol Pretreatment in Dogs

**DOI:** 10.3390/vetsci8070129

**Published:** 2021-07-12

**Authors:** Jennifer Davis, Anthea L. Raisis, Claire R. Sharp, Rachel E. Cianciolo, Steven C. Wallis, Kwok M. Ho

**Affiliations:** 1 School of Veterinary Science, Murdoch University, Murdoch, WA 6150, Australia; a.raisis@murdoch.edu.au (A.L.R.); C.Sharp@murdoch.edu.au (C.R.S.); kwok.ho@health.wa.gov.au (K.M.H.); 2 Centre for Terrestrial Ecosystem Science and Sustainability, Harry Butler Institute, Murdoch University, Murdoch, WA 6150, Australia; 3 Department of Veterinary Biosciences, The Ohio State University, Columbus, OH 43210, USA; Cianciolo.14@osu.edu; 4 University of Queensland Centre for Clinical Research, Brisbane, QLD 4029, Australia; s.wallis@uq.edu.au; 5 Department of Intensive Care Medicine, Royal Perth Hospital, Perth, WA 6000, Australia; 6 Medical School, University of Western Australia, Perth, WA 6009, Australia

**Keywords:** acute kidney injury, biomarkers, coagulation, ischemia-reperfusion injury, shock

## Abstract

Resveratrol has been shown to preserve organ function and improve survival in hemorrhagic shock rat models. This study investigated whether seven days of oral resveratrol could improve hemodynamic response to hemorrhage and confer benefits on risk of acute kidney injury (AKI) without inducing coagulopathy in a canine model. Twelve greyhound dogs were randomly allocated to receive oral resveratrol (1000 mg/day) or placebo for seven days prior to inducing hemorrhage until a targeted mean blood pressure of ≤40 mmHg was achieved. AKI biomarkers and coagulation parameters were measured before, immediately following, and two hours after hemorrhage. Dogs were euthanized, and renal tissues were examined at the end of the experiment. All investigators were blinded to the treatment allocation. A linear mixed model was used to assess effect of resveratrol on AKI biomarkers and coagulation parameters while adjusting for volume of blood loss. A significant larger volume of blood loss was required to achieve the hypotension target in the resveratrol group compared to placebo group (median 64 vs. 55 mL/kg respectively, *p* = 0.041). Although histological evidence of AKI was evident in all dogs, the renal tubular injury scores were not significantly different between the two groups, neither were the AKI biomarkers. Baseline (pre-hemorrhage) maximum clot firmness on the Rotational Thromboelastometry (ROTEM^®^) was stronger in the resveratrol group than the placebo group (median 54 vs. 43 mm respectively, *p* = 0.009). In summary, seven days of oral resveratrol did not appear to induce increased bleeding risk and could improve greyhound dogs’ blood pressure tolerance to severe hemorrhage. Renal protective effect of resveratrol was, however, not observed.

## 1. Introduction

Perioperative hemorrhage is a major complication in surgical patients, resulting in increased morbidity and mortality [1,2,3,4]. Severe hemorrhage can lead to severe hypotension resulting in reduced vital organs perfusion and oxygenation [5]. Ischemia followed by reperfusion—also known as ischemia-reperfusion (I-R) injury—will induce formation of free radical oxygen species, inflammatory mediators, and toxic metabolites which can cause organ injury [6,7,8]. Indeed, I-R injury is one of the main causes of perioperative acute kidney injury (AKI) [9,10]. AKI is common in patients undergoing major surgery and can contribute to increased perioperative mortality [11,12,13,14,15]. As such, strategies capable of attenuating I-R injury due to severe hemorrhage are both clinically relevant and desirable.

Resveratrol is a naturally occurring polyphenol and administrating resveratrol in large doses parenterally has been shown to preserve organ function and improve survival in hemorrhagic shock rat models [6]. Resveratrol can activate expression of the Silent Information Regulator (SIRT1) gene to produce situin-1 protein which can inhibit pro-inflammatory mediators, enhance anti-oxidant pathways, and improve mitochondrial function. In addition, it also has an agonist effect at estrogen receptors [6,7,8,16]. These effects may explain why administering intraperitoneal or parenteral resveratrol as a resuscitation agent could improve blood pressure in rats with decompensated hemorrhagic shock [6,8]. Although rodent models are appropriate to investigate the mechanistic benefits of resveratrol, confirmation of resveratrol’s benefits in large-animal models is needed before it can be safely tested on humans in a clinical setting [17,18]. Previous experimental studies have primarily used a single large dose of resveratrol during or after hemorrhage [6,8]. Because sterile preparation of parenteral resveratrol is not commercially available, translating this treatment strategy to patients at risk of hemorrhage remains difficult.

It is possible that oral resveratrol, taken for a prolonged period of time prior to hemorrhage, may achieve a similar benefit as parenteral resveratrol in hemorrhage. If this is the case, this strategy would be more clinically applicable for patients who are at risk of massive blood loss in elective major surgery. Another concern about the use of resveratrol in perioperative patients is its potential antiplatelet action [19,20]. Currently no safety data exists to guide clinicians whether resveratrol should be ceased prior to major surgery to avoid increased risk of bleeding.

We hypothesized that seven days of oral resveratrol treatment would improve hemodynamic tolerance to induced bleeding. Using a canine model, we aimed to investigate whether resveratrol, administered orally for seven days prior to anesthesia, could improve blood pressure tolerance due to induced hemorrhage. Our secondary objective was to investigate whether resveratrol could protect against AKI without inducing adverse effect on the coagulation.

## 2. Materials and Methods

### 2.1. Animals and Selection Criteria

Twelve donated adult entire male dogs were included in the study. The dogs were retired racing greyhounds, surrendered by their owners to be used as terminal blood donors. Physical examination, renal ultrasonography, urinalysis, complete blood count, serum creatinine (SCr), blood urea nitrogen, serum albumin concentration, platelet closure time (PCT), and Rotational Thromboelastometry (ROTEM^®^ delta, Tem International GmbH, Munich, Germany) for all dogs were within reference intervals for adult Greyhounds [21]. Ethics approval was granted by the Murdoch University Animal Ethics Committee (permit number R2726/15) and the dogs cared for in accordance with the Australian code for the care and use of animals for scientific purposes.

### 2.2. Resveratrol Administration

Dogs were randomly allocated to receive either no resveratrol supplementation (C-control group; *n* = 6) or seven days of 1000 mg (>10 mg/kg) per day of micronized trans-resveratrol (Micronized Resveratrol Micro500, Harmoni-T, Las Vegas, NV 89119, USA) orally (R-resveratrol group; *n* = 6), via a computer-generated random number sequence (Excel, Microsoft Corporation, Redmond, WA, USA). The final dose of resveratrol was administered in the morning of anesthesia. Serum and urine supernatant collected during the enrolment screening process for all dogs was divided into aliquots and stored at −80 °C as “baseline” samples for subsequent analysis of creatinine, protein, gamma-glutamyl transferase (GGT), and AKI biomarker concentrations. Animals in both groups were cared for in the same premises, under the same conditions, for seven days prior to anesthesia. Throughout this period, water was provided ad lib, and dogs were fed an intestinal health diet (Hills I/D) twice daily. All dogs received a deworming prophylactic medication (Popantel^®^, Jurox Animal Health, Rutherford, NSW, Australia) at the time of enrolment into the study. Dogs in both groups were examined daily by a veterinarian to ensure maintenance of good health, and any abnormalities detected on physical examination recorded.

### 2.3. Anesthesia

For all dogs, food was removed at least eight hours prior to the procedure but access to water provided until premedication. Dog were anesthetized by a veterinary anesthesiologist (AR) using a standardized protocol. Premedication with methadone 0.3 mg/kg (Ilium Methadone 10 mg/mL, Troy Laboratories, Glendenning, NSW, Australia) IM was performed 30 min prior to induction of general anesthesia with alfaxalone (Alfaxan injection 10 mg/mL, Jurox, Glendinning, NSW, Australia) 2.2–3.2 mg/kg intramuscularly. Endotracheal intubation was used to maintain anesthesia with isoflurane (I.S.O., Veterinary Companies of Australia Pty Ltd, Kings Park, NSW, Australia) in 30% oxygen through a circle rebreathing system. End tidal isoflurane was maintained at 1.3–1.4%. Infusion of a balanced isotonic crystalloid solution (Compound Sodium Lactate, Baxter Healthcare, Toongabbie, NSW, Australia) was provided at 10 mL/kg/hour IV throughout anesthesia, along with IV fentanyl (Fentanyl injection 50 µg/mL, AstraZeneca, Macquarie Park, NSW, Australia) 2 µg/kg/hour. Arterial carbon dioxide tension was maintained between 35 and 45 mmHg by provision of mechanical ventilation by an anesthesia workstation (Datex-Ohmeda S/5 Aespire Anaesthesia Machine, GE Healthcare, Chicago, IL, USA). Active warming maintained esophageal temperature between 36.0 and 38.0 °C.

### 2.4. Instrumentation Procedures

Instrumentation procedures were performed within the first 60 min of anesthesia. Dogs were positioned in dorsal recumbent position during this period. The cranial vena cava was cannulated via the right jugular vein, using a 14-gauge 13 cm cannula, to facilitate collection of venous blood and injection of lithium chloride. To allow measurement of arterial blood pressure and cardiac output (Qt) via the lithium dilution technique, and to facilitate controlled hemorrhage, a 14-gauge 9 cm cannula was placed in the left femoral artery. Prior to surgical exposure of the femoral artery and cannula placement, a femoral nerve block was performed using 0.1 mL/kg bupivacaine (Bupivacaine hydrochloride 5.0 mg/mL, Pfizer, Sydney, NSW, Australia). Non-distensible tubing filled with heparinized saline connected the arterial cannula to an electronic pressure transducer (DTX Plus, Argon Critical Care Systems, Singapore) for measurement of arterial blood pressure using a multi-parameter monitor (Surgivet V9203; Smiths Medical, Minneapolis, MN, USA). Before anesthesia of each animal, the transducer was calibrated using a mercury manometer (at 40, 80 and 120 mmHg). Once connected, the transducer was placed level with the manubrium of the sternum (i.e., approximate level of the right atrium) and zeroed to atmospheric pressure. Hourly during the study (just prior to each data collection interval), damping factor was subjectively assessed via a rapid flush test. Lack of baseline drift was confirmed at the end of each anesthetic by ensuring pressure read zero upon re-opening the transducer to the atmosphere. An 8 Fr 55 cm Foley urinary catheter was inserted into the bladder for collection of urine samples and measurement of urine output (UOP).

### 2.5. Experimental Design

**Figure 1:**
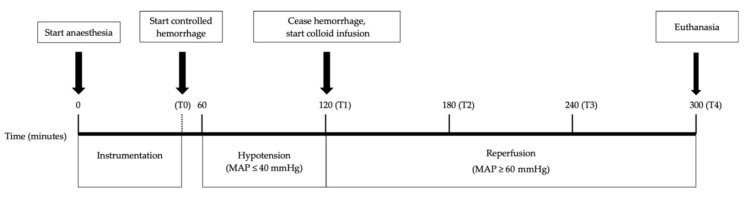
Timeline of the pressure-targeted hemorrhage model performed in 12 adult male Greyhounds. MAP = mean arterial pressure. T0–T4 were time points at which cardiac output was measured, and blood (arterial and venous) and urine samples were collected.

This study utilized a predefined blood pressure (mean arterial pressure-MAP of ≤40 mmHg) target to assess whether the volume of blood loss needed to achieve such target was different between the two groups (see Figure 1). Following instrumentation and collection of baseline samples (T0), bleeding was commenced by removing blood from the femoral artery cannula until MAP fell to ≤40 mmHg and remained at that level for 60 mins. After the 60-min hypotensive period was completed, intravenous fluid resuscitation was initiated using 20 mL/kg of succinylated gelatine solution 4% (Gelofusine^®^, B. Braun, Bella Vista, NSW, Australia) at 1200 mL/h (without crystalloid boluses) until MAP was ≥60 mmHg. The dogs then underwent a further three hours of anesthesia with their MAP maintained at ≥60 mmHg, by infusing more Gelofusine^®^ if required. Approximately about five hours following induction of anesthesia, the animals were euthanized using pentobarbitone (Lethabarb Euthanasia Injection 300 mg/mL, Virbac, Milperra, NSW, Australia) 150 mg/kg IV. The total amount of blood volume needed to achieve and maintain the 60 min hypotensive period and the amount of colloid fluid needed to achieve normotension subsequently were recorded.

### 2.6. Data Collection

Data collection was performed at T0 (baseline: 60 min after induction of anesthesia, following instrumentation, prior to ischemia), T1 (60 min after MAP first reached ≤40 mmHg, prior to reperfusion), T2 (60 min after commencing reperfusion), T3 (120 min after reperfusion commenced), and T4 (180 min after reperfusion commenced).

#### 2.6.1. Cardiovascular Parameters

At each time point, the mean of five consecutive MAP measurements was calculated. A blood gas analyzer and co-oximeter (ABL 725, Radiometer, Copenhagen) was used to measure blood gas variables (oxygen tension, carbon dioxide tension, bicarbonate concentration, calculated base excess, oxygen saturation) on arterial and central venous blood collected into heparinized syringes (PICO syringe, Radiometer, Copenhagen). These samples were analyzed immediately following collection. The same analyzer was used to measure hemoglobin and sodium concentration in central venous blood, necessary for the Qt measurement using lithium dilution. The lithium dilution technique, previously validated for Qt measurement in dogs, was performed at each time interval [22]. The mean of two consecutive Qt measurements that were not different by >10% was used for analysis. The calculation of the oxygen extraction ratio (OER) is described in Table 1. OER reflects the balance between systemic oxygen delivery and demand, and an OER greater than 0.5 in anesthetized dogs implied the presence of anaerobic metabolism according to previous studies [23,24,25].

#### 2.6.2. Renal Parameters

##### Urine Sediment Examination

At each time point urine bag was emptied into a sterile collection pot for UOP measurement, and urine specific gravity and dipstick analyses (Multistix^®^, Siemens, Erlangen, Germany). Urine samples were centrifuged at 339 g for five minutes. At each time interval, urine sediment in the urine was examined using light microscopy at low (×100) and high (×400) power. A minimum of ten high power fields were examined for presence of blood cells, epithelial cells, and granular and hyaline casts. Urine supernatant was divided into multiple aliquots and stored at −80 °C for later analysis of creatinine, protein, GGT, and biomarker concentrations.

##### Kidney Light Microscopy

Kidneys were removed immediately following euthanasia, sectioned and stored in 10% formalin. Additionally, small cubes of cortex (approximately 2 to 3 mm^3^) were placed into 3% glutaraldehyde (Koch-Light, Johannesburg, South Africa) for transmission electron microscopic (TEM) evaluation. Samples were processed routinely by the Comparative Pathology and Mouse Phenotyping Shared Resource (The Ohio State University, Columbus, OH, USA). Samples were embedded in paraffin, sectioned at 3µm thickness and stained with hematoxylin and eosin and periodic acid-Schiff. One veterinary pathologist, blinded to group allocation and AKI biomarker concentrations, examined sections of both kidneys by light microscopy and counted the number of injured tubules per 200× field. Tubular injury was defined as loss of the apical brush border, denudation of tubular basement membranes, singly dead tubular epithelial cells and tubules with intra-luminal detached cells/cellular debris. Twenty randomly selected cortical fields were assessed, and the average number of injured tubules per field was calculated for each specimen. Based on these quantitative data, the samples were then categorized into groups of normal (no injured tubules), minimal (mean tubular injury ≤0.5), mild (mean tubular injury >0.6 but ≤1) and moderate (mean tubular injury >1 but ≤2) histologic injury.

##### Kidney Transmission Electronic Microscopy (TEM)

Due to resources constraint, the glutaraldehyde-fixed tissue from only 4 dogs (2 control and 2 treated) were processed for electronic microscopy. After post-fixation in 1% osmium tetroxide, the specimens were serially dehydrated, infiltrated in an acetone/epoxy plastic, and embedded in plastic. The plastic blocks were sectioned to a silver-grey interference color (55–60 nm) and placed on copper mesh grids. The sections were stained with filtered lead citrate/sodium citrate solution (Electron Microscopy Sciences, Inc., Hatfield, PA, USA). Grids were imaged on a JEOL JEM-1400 TEM (JEOL USA, Inc., Peabody, MA, USA) and representative images were photographed with an Olympus SIS Veleta 2K camera (Olympus Soft Imaging Solutions GmbH, Muenster, Germany.

##### Renal Biomarkers

At baseline, T0, T1, and T3; aliquots of plain serum and urinary supernatant were stored at −80 °C for measurements of SCr and AKI biomarkers within three months.

Urinary creatinine, protein, and GGT concentrations; and SCr concentration, were measured using the same biochemistry analyzer (Cobas Integra 400 plus, Roche Diagnostics) that was calibrated prior to each use, with two control samples included in each run. Concentrations of AKI biomarkers; clusterin, cystatin C, kidney injury molecule 1 (KIM-1), monocyte chemoattractant protein 1 (MCP-1), and neutrophil gelatinase-associated lipocalin (NGAL), were measured in the urine and serum samples from each dog at baseline, T0, T1, and T2 using a bead-based multiplexed immunoassay (MILLIPLEX™ MAP Canine Kidney Toxicity Expanded Magnetic Bead Panel 1, Merck Millipore, Burlington, MA, USA). Urinary samples were diluted to 1:2, and serum samples to 1:10, using assay buffer prior to analysis. All samples were analyzed in duplicate. The assays were performed according to manufacturer instructions. Briefly, samples were incubated with a solution of antibody-immobilized magnetic beads (for each of the five biomarkers previously listed) for two hours at room temperature. The plate was then washed using a magnetic plate washer (Bio-Plex^®^ Pro II Wash Station, Bio-Rad, Hercules, CA, USA), detection antibodies added, and incubated at room temperature for one hour. Streptavidin-Phycoerythrin was added to the detection antibodies, prior to a further 30 min period of incubation at room temperature. The plate was then washed, and beads re-suspended with drive fluid (MAGPIX^®^ Drive Fluid, Merck Millipore, Burlington, MA, USA). The plate was analyzed five minutes later using a multiplex reader (Bio-Plex^®^ MAGPIX™ Multiplate Reader, Bio-Rad, Hercules, CA, USA) with xPONENT^®^ software. The Median Fluorescent Intensity data was analyzed using a 5-parameter logistic curve to calculate analyte concentrations in each sample. If the coefficient of variance between two duplicates was >15% the sample was re-analyzed on a subsequent plate. If the analyte concentration of a sample was above the upper limit of quantification (ULOQ) the sample was diluted until the biomarker concentration fell within the measurable range of the assay. If the analyte concentration of a sample was below the lower limit of quantification (LLOQ) the sample was reanalyzed on a subsequent plate using undiluted sample. Any result still falling below the LLOQ was allocated the same value as the LLOQ in order to allow statistical analysis. When serum samples were analyzed using the multiplex assay, a serum diluent (Serum Matrix, Merck Millipore, Burlington, MA, USA) was added to wells containing the Standards and Controls in place of assay buffer in order to mitigate any matrix effects.

#### 2.6.3. Coagulation Parameters

Central venous blood was obtained from the jugular vein catheter at each time point for assessment of coagulation. At T0, T1, and T2, blood samples for coagulation assay were transferred into blood tubes containing 3.2% buffered sodium citrate with a blood: anticoagulant ratio of 9:1. Tubes were gently agitated to ensure even mixing of anticoagulant. Platelet closure time (PCT) was measured immediately, and blood was stored at room temperature for Rotational Thromboelastometry analysis (ROTEM^®^ delta, Tem International GmbH, Munich, Germany) 30 min after collection.

The Platelet Function Analyzer-100 (Dade Boehring Inc., Deerfield, IL, USA), with collagen and adenosine diphosphate cartridges, was used to measure PCT in duplicate within 10 min of sample collection. Analysis was immediately repeated if the coefficient of variation was greater than 15%. ROTEM^®^ was performed according to the manufacturer’s instructions using both InTEM and ExTEM. Each profile was run for at least one hour following initiation. Data recorded for the InTEM and ExTEM profiles included clotting time (CT), clot formation time (CFT), alpha angle, maximum clot firmness (MCF) and lysis index at 60 min (LI60). Peak thrombin generation (normal range 243–507 nM) and area under the thrombin generation curve (AUC: normal range 1500–2835 nM × min) were measured by the Calibrated Automated Thrombogram using Thrombinoscope^®^ (Stago^TM^, Asnières sur Seine, France).

Total concentrations of resveratrol in plasma and qualitative investigation of its metabolites (glucuronide and sulphate) both prior to the administration and after 7 days of resveratrol treatment (or control) were measured by the UHPLC-MS/MS on a Shimadzu Nexera2 UHPLC system coupled to a Shimadzu 8030+ triple quadrupole mass spectrometer (Kyoto, Japan) [26].

### 2.7. Data Analysis

A sample size calculation was performed using data from a previous study by Holthoff et al. [27] in which the benefits of resveratrol on septic AKI were quantified by histological changes in a murine model (*n* = 6). A sample size of 6 dogs per group would be needed assuming a difference in histology score of 1.2 between the treatment and control groups, with a standard deviation in the scores of 0.6, in order to achieve 80% power while taking an alpha-value <0.05 as significant. Due to the small sample size of this study, a non-parametric Mann–Whitney test was used to compare the outcomes between the two treatment groups. The changes in MCF on the ROTEM between the two groups was analyzed by adjusting for volume of blood loss in each animal using a linear mixed model. All analyses were two tailed using SPSS for Windows (version 23, IBM, Armonk, NY, USA) and MedCalc Statistical Software (version 18.11.3, Ostend, Belgium). A *p*-value < 0.05 was taken as significant, and no adjustment was made for multiple statistical testing in this study.

## 3. Results

Six dogs were included in each group. Exact age of each dog was unknown, but veterinary examination suggested all dogs were adult (2–7 years). The body weights of the dogs were not statistically different between the two groups (median [range] for the control group was 30.9 kg (30.1–32.5), and for treatment group was 32.6 kg (27.9–34.0); *p* = 0.441). All dogs had a lean body condition (score 3/9), appropriate for trained racing dogs of this breed. No adverse effects of oral resveratrol were noted, and all dogs completed the study. There was no significant difference in renal and coagulation parameters between the two groups prior to initiation of resveratrol treatment (Table 2 and Table 3), but there was a suggestion that seven days of resveratrol treatment could improve clot strength compared to the control prior to induction of hemorrhage (InTEM MCF 54 vs. 43 mm respectively; *p* = 0.009)(Table 3). No resveratrol and its metabolites were detected in the control dogs. Among the six dogs treated with resveratrol, only one had a detectable level of resveratrol at a concentration < 50 mg/mL.

### 3.1. Cardiovascular Results

**Figure 2:**
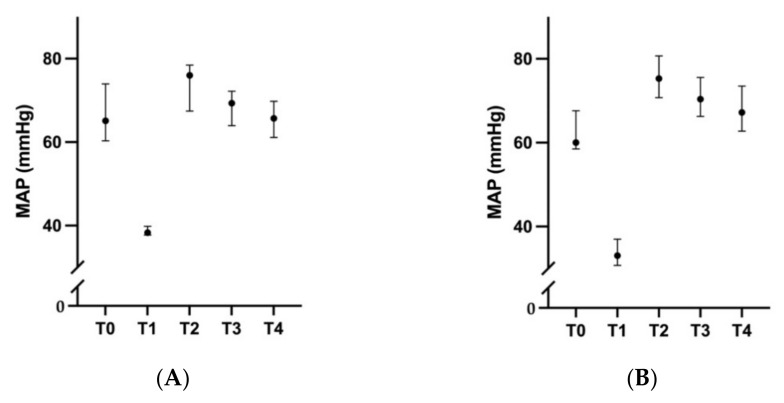
Changes in mean arterial pressure (MAP) over time in anesthetized greyhounds subjected in a pressure-targeted hemorrhage model. Values are median and interquartile range. (**A**) Control group (*n* = 6). (**B**) Resveratrol pretreated group (*n* = 6).

MAP over time are summarized in Figure 2. The median time to induce hemorrhage to achieve a MAP ≤ 40 mmHg was within 4 min (interquartile range [IQR] 2.75–8.75) in the treatment group and 5 (3–7.75) minutes in the control group which were not statistically different (*p* = 0.939). A significantly larger amount of blood was, however, withdrawn to achieve and maintain this MAP target for 60 min for the dogs treated with resveratrol compared to control (median 63.8 vs. 54.9 mL respectively, *p* = 0.041), with a slightly lower MAP at end of one-hour of hemorrhage in the resveratrol group (33 vs. 39 mmHg respectively, *p* = 0.041). Arterial and venous pH were also significantly lower in the treatment group versus control group at end of one-hour of hemorrhage (arterial 7.24 vs. 7.35, *p* = 0.011; venous 7.23 vs. 7.32, *p* = 0.047 respectively). The other blood gas and hemodynamic parameters between the two groups were not significantly different (Table 4 and Table 5). Time for the MAP to return to ≥ 60 mmHg following cessation of induced bleeding and initiation of Gelofusine^®^ fluid resuscitation was not different between the two groups (R group: median 17, IQR 12.5–25 vs. C group: 14, IQR 11.3–17.3; *p* = 0.571). No additional fluid administration was required in any dog in order to maintain MAP > 60 mmHg during the subsequent three-hour reperfusion period. There were also no significant differences in the arterial and venous blood gases including OER between the two groups during the experiment.

### 3.2. Renal Outcomes

Renal data from one dog in the resveratrol group were excluded from further analysis due to identification of pre-existing renal inflammatory disease on histological examination of the kidney tissue. Except for the urine KIM- 1 (*p* = 0.536), all kidney injury parameters increased significantly (*p*-values < 0.05) within 2 h after induced hemorrhage. The risks of AKI—defined according to the IRIS criteria [22]—were not significantly different (*p* = 0.844) between the resveratrol (no AKI = 3 dogs, AKI grade 2 = 3 dogs) and control groups (no AKI = 1 dog, AKI grade 1 = 1 dog, AKI grade 2 = 4 dogs). All serum and urinary AKI biomarkers were also similar between the two groups (Table 2).

### 3.3. Coagulation Outcomes

The clotting time became prolonged (*p* = 0.008) and MCF was weakened in both InTEM (*p* = 0.001) and ExTEM (*p* = 0.001) in the study dogs after hemorrhage. In addition to having a better clot firmness (or MCF) prior to hemorrhage, dogs treated with resveratrol also had a higher peak thrombin generation potential at 1-h after hemorrhage (Table 3). MCF on the InTEM appeared to remain better in the resveratrol group during the period of hemorrhage after adjusting for volume of blood withdrawn in each animal in the linear mixed model analysis (Table 6).

### 3.4. Renal Histopathology

**Figure 3:**
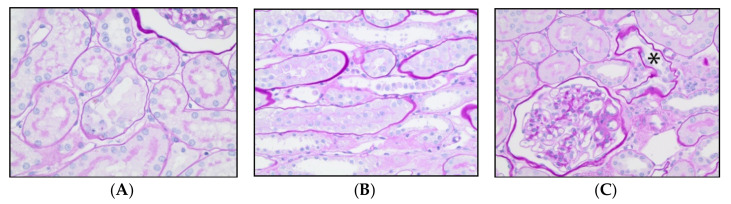
(**A**) Photomicrograph from a control dog with mild acute tubular epithelial injury, characterized by loss of the brush border and simplification of the tubular epithelium. There are 2 sloughed necrotic cells within the lumen. (**B**) Photomicrograph from a treated dog with mild acute tubular epithelial injury characterized by the presence of many sloughed epithelial cells within a tubular lumen. (**C**) Photomicrograph from a treated dog that had moderate acute tubular epithelial injury, characterized by simplification and attenuation of tubular epithelium (asterisk) that was more frequently observed than in dogs with mild injury. Periodic acid Schiff, 40 × magnification.

Notably, severe tubular injury (evidence of ischemic coagulative necrosis) was not identified in any specimen. In the 20 randomly selected 200× fields of cortex (due to the lack of lesions in the deeper regions of the renal parenchyma) for each kidney examined, there was evidence of injury (loss of brush border, singly dead cells, sloughed cellular debris) in the study animals. Both control and resveratrol treated dogs had normal to mildly injured tubules (Figure 3A,B). One resveratrol-treated dog had evidence of moderate tubular injury (Figure 3C).

### 3.5. Renal Transmission Electron Microscopy (TEM)

**Figure 4:**
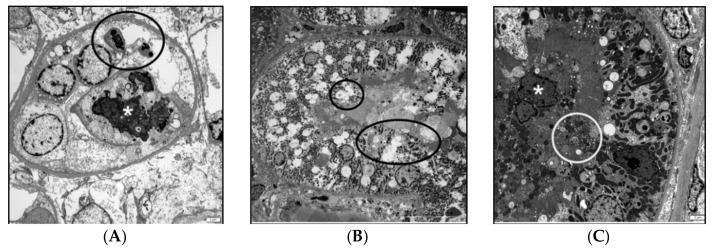
(**A**) Transmission electron micrograph from a control dog that had minimal acute tubular epithelial injury in the histology sample. The sample submitted for TEM showed sloughed cellular debris (circled) and electron dense cytoplasmic material (mineralization) (asterisk). There is denuded tubular basement membrane. Bar is 2 micron. (**B**) Transmission electron micrograph from a treated dog that had mild acute tubular epithelial injury in the histology sample. There is loss of the apical brush border of multiple cells (circled) whereas neighboring cells maintain intact brush borders. Bar is 20 micron. (**C**) Transmission electron micrograph from a control dog that had minimal injury in the histology sample. There is loss of the apical brush border (circled) as well as sloughed cells within the tubular lumen (asterisk). Bar is 2 micron.

Samples from two control dogs and two treated dogs were further evaluated using TEM. All four dogs had evidence of loss of the apical brush border, cellular swelling and intraluminal cellular fragments (Figure 4). *Proximal tubules* were most frequently damaged but distal convoluted tubules were occasionally affected. Ultra-structurally, there were no obvious differences between the two dogs in each study group (although this was not formally tested statistically because of the small sample size of this sub-group). Dogs that had minimal injury in the histology specimen had similar ultrastructural lesions on the TEM as dogs that had mild and moderate histologic lesions.

## 4. Discussion

This study showed that seven days of oral resveratrol treatment in greyhound dogs improved their blood pressure tolerance to induced hemorrhage; that is, treated animals required a larger amount of blood loss to develop the same degree of hypotension. There was also a signal to suggest that resveratrol might improve clot strength and thrombin generation. The potential renal protective effect of resveratrol was, however, not observed both in terms of biochemical and histological assessments. These findings are clinically relevant and require further discussion.

First, a blood pressure targeted hemorrhage model was used in this study in an attempt to assess whether resveratrol could improve the maintenance of blood pressure during hemorrhage. Whether this was a better model than a model that induces a fixed amount of blood loss remains uncertain [28]. The volume of blood loss to achieve the predetermined MAP in our control animals (median 54.9, range 42.5–58.9 mL/kg) was consistent with the results of another study (53 mL/kg, 95% confidence interval 48–57) [29]. As such, the greater amount of blood loss needed to achieve and maintain the same degree of hypotension (and OER in the central venous blood) in the resveratrol-treated dogs (median 63.8, range 57.8–78.6 mL/kg) suggests that oral resveratrol pretreatment may have genuinely assisted the dogs in maintaining a better blood pressure during acute bleeding. This beneficial effect of resveratrol may theoretically, at least in part, be related to activation of SIRT1 gene, its estrogen agonist effect, or interactions with other targets or receptors that are relevant to pathogenic process of I-R injury [6,7,8]. Because we could not detect resveratrol levels in most blood samples of our resveratrol-treated dogs, the precise mechanisms how resveratrol can help to maintain the blood pressure remains unclear, but modification of gut microbiota is one possibility [30]. Nonetheless, the dogs in the resveratrol group did have a lower MAP and pH in the arterial and venous blood at the end of 60-min of hemorrhage. Repeating the study with a larger sample size, using a volume-guided hemorrhagic model using preset volume of blood loss, and extending the study duration to include survival time and effect on gut microbiota would be helpful to confirm the benefits of resveratrol, and clarify the mechanisms through which oral resveratrol pretreatment improves cardiovascular tolerance to hemorrhage.

Second, all study dogs had an increase in many serum and urinary AKI biomarkers induced hemorrhage; and these changes were associated with histopathological evidence of AKI. This was not surprising as a reduction in blood volume and pressure would certainly compromise renal blood flow, triggering a reduction in glomerular filtration rate and induction of renal ischemia [31]. As such, the hemorrhagic model used in our study was sensitive enough to induce bleeding-related AKI [32]. Despite this, there were no significant difference in all the renal biomarkers and histological changes between the resveratrol and control groups. This negative result could be explained by a number of reasons. First of all, a much lower dose (per body weight) of resveratrol was used in this study and outcomes were assessed in a shorter time frame than in the rat models in previous studies [6,7,8,16,27,33]. Furthermore, a small sample size could undermine the statistical power of this study. Of the dogs that were treated with resveratrol, 40% (2/6 dogs) developed stage-1 AKI (defined by an increment in SCr > 26.4 µmol/L) compared to 100% of the dogs in the control group, even though this difference did not reach statistical significance. Another possibility is that resveratrol might have different renal effects in different animal species. To the best of our knowledge, using resveratrol to prevent AKI during hemorrhage in dogs (in contrast to rats [16]) has not been assessed previously. Some types of intravenous fluid used for resuscitation can also have an adverse effect on the kidneys. Although the same types and similar quantities of intravenous fluid (Compound Sodium Lactate for maintenance at 10 mL/kg/hour and Gelofusine^®^ to restore normal blood pressure: see Table 4) were used for both groups in this study, it is possible that resveratrol’s renal protective effect—if there is one—was confounded or overwhelmed by a potential harmful or beneficial effect of Gelofusine^®^ on the kidneys. Previous studies have shown that using Gelofusine^®^ for resuscitation was less likely to induce AKI compared to the older forms of intravenous starches in humans [34], but recent animal studies have shown that Gelofusine^®^ may induce AKI compared to crystalloids or fresh whole blood [35]. As such, it would be better to use crystalloid fluid alone during the reperfusion period in similar experiments in the future.

An interesting and important finding of this study was the positive association between resveratrol, clot firmness and thrombin generation. A previous study has showed that resveratrol could help to preserve platelet function in stored blood [36]. Conversely, resveratrol has also been shown to induce platelet dysfunction and coagulation derangements in a few other experimental studies [19,20,37,38]. Given hemostasis is a paramount outcome in any hemorrhagic situations, the effect of resveratrol on bleeding tendency requires further investigation.

Finally, we need to acknowledge the limitations of this study. The circumstances of how our study animals were recruited had limited our ability to address the important issue of survival time after hemorrhage. It is important to note that race-trained greyhounds are highly adapted to anaerobic tissue metabolism during exercise, modulating their cardiovascular, hemostatic and homeostatic responses to hemorrhage [39,40]. Prolonged anesthesia (5 h in this study) might have interfered with the normal cardiovascular compensatory responses to bleeding. In addition, pure μ-agonist opioids, such as the fentanyl used in this study, are known to cause venous and arterial blood wall relaxation, via endothelium and opioid-receptor independent mechanisms in various species [41,42]. Similarly, isoflurane also has a dose-dependent vasodilatory and negative inotropic effect, rendering our study animals more likely to develop hypotension after hemorrhage [43,44]. Nonetheless, a parallel-arm randomized-controlled design should have balanced the confounding effects of these factors.

## 5. Conclusions

This exploratory study showed that seven days of oral resveratrol treatment prior to bleeding improved greyhound dogs’ blood pressure stability in response to severe hemorrhage and possibly also coagulation profiles compared to no resveratrol treatment. A renal protective effect of resveratrol in hemorrhage was, however, not observed. An adequately-powered study using a volume-targeted hemorrhage model assessing the benefits of resveratrol, including survival time, is needed before this dietary supplement can be recommended prior to exposure to hemorrhage.

## Figures and Tables

**Table 1:** Calculation of oxygen extraction ratio (OER).

Calculated Variable	Formula
BSA (m^2^)	10.1 × (bodyweight in grams^2/3^) × 10^−4^
Cardiac index (L minute^−1^ (m^2^)^−1^)	Qt ÷ BSA
CaO_2_ (mL L^−1^)	(1.36 × SaO_2_ × Hb) + (0.003 × PaO_2_) × 10
DO_2_I (mLO_2_ min^−1^ (m^2^)^−1^)	Cardiac index × CaO_2_
CcvO_2_ (mL L^−1^)	(1.36 × ScvO_2_ × Hb) + (0.003 × PcvO_2_) × 10
VO_2_I (mLO_2_ min^−1^ (m^2^)^−1^)	Cardiac index × (CaO_2_ − CcvO_2_)
OER	VO_2_I ÷ DO_2_I

BSA = body surface area; CaO_2_ = arterial oxygen content; CcvO_2_ = central venous oxygen content; DO_2_I = oxygen delivery index; Hb = hemoglobin concentration; PaO_2_ = arterial oxygen tension; PcvO_2_ = central venous oxygen tension; Qt = cardiac output; SaO_2_ = arterial oxygen saturation; ScvO_2_ = central venous oxygen saturation; VO_2_I = oxygen consumption index.

**Table 2:** Renal parameters of anesthetized greyhound dogs before and after induced hemorrhage (*n* = 11).

Variables	Resveratrol Treated (*n* = 5)	Control (*n* = 6)	*p*-Value ^#^
Serum creatinine, µmol/L:*			
-Pre-treatment	129 (121–146)	118 (105–128)	0.126
-Pre-hemorrhage	125 (113–136)	105 (99–120)	0.126
-1 h post-hemorrhage	145 (133–155)	133 (128–151)	0.329
-2 h post-resuscitation	155 (145–185)	154 (130–167)	0.537
Serum cystatin:			
-Pre-treatment	49 (36–56)	45 (36–58)	0.999
-Pre-hemorrhage	52 (41–63)	46 (41–54)	0.537
-1 h post-hemorrhage	64 (46–69)	56 (49–72)	0.931
-2 h post-resuscitation	47 (32–55)	39 (31–47)	0.537
Serum NGAL:			
-Pre-treatment	8.4 (4.6–12.8)	8.8 (4.8–12.9)	0.999
-Pre-hemorrhage	9.4 (7.7–20.2)	10.8 (8.5–13.1)	0.931
-1 h post-hemorrhage	12.4 (11–22.9)	14.9 (12.3–22.8)	0.662
-2 h post-resuscitation	12.1 (7.8–19.5)	11.2 (9.9–16.9)	0.999
Serum KIM-1:			
-Pre-treatment	59 (51–94)	75 (42–101)	0.73
-Pre-hemorrhage	71 (54–87)	74 (53–90)	0.999
-1 h post-hemorrhage	54 (46–68)	64 (48–75)	0.537
-2 h post-resuscitation	39 (35–45)	42 (32–47)	0.931
Urine clusterin:			
-Pre-treatment	219 (146–617)	250 (83–921)	0.999
-Pre-hemorrhage	299 (134–348)	72 (24–186)	0.082
-1 h post-hemorrhage	318 (147–1315)	368 (49–554)	0.999
-2 h post-resuscitation	2242 (1275–3769)	1381 (727–4443)	0.537
Urine cystatin:			
-Pre-treatment	3.6 (2.9–4.3)	3.2 (2.1–3.8)	0.42
-Pre-hemorrhage	3.8 (2.5–5.4)	3.7 (1.8–5.7)	0.999
-1 h post-hemorrhage	4.0 (2.7–5.9)	5.0 (1.7–7.5)	0.999
-2 h post-resuscitation	8.7 (8.0–10.8)	9.1 (7.3–10.6)	0.792
Urine NGAL:			
-Pre-treatment	0.5 (0.3–3.7)	1.5 (0.1–6.2)	0.999
-Pre-hemorrhage	1.2 (0.3–3.0)	0.9 (0.6–3.3)	0.931
-1 h post-hemorrhage	1.1 (0.3–2.3)	0.8 (0.6–2.8)	0.841
-2 h post-resuscitation	25.6 (18.1–30.1)	19.7 (16.2–24.7)	0.429
Urine KIM-1:			0.052
-Pre-treatment	50 (8–119)	139 (109–279)	
-Pre-hemorrhage	22 (11–138)	82 (44–184)	0.247
-1 h post-hemorrhage	18 (12–223)	137 (38–415)	0.286
-2 h post-resuscitation	49 (38–75)	57 (36–115)	0.662
Urine GGT to creatinine ratio:			
-Pre-treatment	21 (4–224)	13 (9–21)	0.841
-Pre-hemorrhage	25 (21–145)	51 (29–80)	0.548
-1 h post-hemorrhage	76 (24–306)	39 (26–285)	0.999
-2 h post-resuscitation	1190 (842–12996)	1299 (420–2500)	0.69
Glomerular injury, %	3.9 (2.4–15.1)	0.6 (0–3.7)	0.082
Tubular injury score	0.30 (0.28–0.89)	0.30 (0.11–0.49)	0.429

^#^*p*-value by non-parametric Mann–Whitney test. All values are median (with interquartile range –IQR unless stated otherwise). Except for the urine KIM-1 (*p* = 0.536), all kidney injury parameters listed in this table differed significantly (*p*-values < 0.05) during the time course of the experiment. * The number of animals developed stage-1 acute kidney injury–with an increment in serum creatinine >26.4 µmol/L-between the resveratrol (*n* = 2, 40%) and control (*n* = 6, 100%) groups appeared different but this was not statistically significant (*p* = 0.061). GGT = gamma-glutamyl transferase; KIM-1 = kidney injury molecule 1; NGAL = neutrophil gelatinase-associated lipocalin.

**Table 3:** Rotational Thromboelastometry (ROTEM^®^) viscoelastic and thrombin generation parameters of the anesthetized greyhound dogs before and after induced hemorrhage (*n* = 9).

Variables	Resveratrol Treated (*n* = 5)	Control (*n* = 4)	*p*-Value ^#^
CT-InTEM:			
-Pre-treatment	198 (167–233)	194 (169–209)	0.73
-Pre-hemorrhage	168 (137–192)	133 (80–169)	0.177
-1 h post-hemorrhage	142 (130–165)	168 (140–174)	0.548
-2 h post-resuscitation	160 (142–200)	183 (168–193)	0.286
CT-ExTEM:			
-Pre-treatment	71 (40–90)	72 (49–98)	0.556
-Pre-hemorrhage	40 (35–60)	86 (31–155)	0.429
-1 h post-hemorrhage	62 (38–76)	101 (65–112)	0.082
-2 h post-resuscitation	76 (69–88)	113 (100–188)	0.016
MCF-InTEM:			
-Pre-treatment	51 (44–56)	48 (43–50)	0.556
-Pre-hemorrhage	54 (47–63)	43 (38–47)	0.009
-1 h post-hemorrhage	38 (33–45)	40 (32–44)	0.841
-2 h post-resuscitation	33 (30–41)	33 (28–37)	0.556
MCF-ExTEM:			
-Pre-treatment	41 (38–51)	40 (33–47)	0.73
-Pre-hemorrhage	52 (47–63)	45 (33–50)	0.052
-1 h post-hemorrhage	38 (34–45)	27 (25–36)	0.052
-2 h post-resuscitation	35 (30–39)	21 (14–32)	0.063
PFA-closure time:			
-Pre-hemorrhage	78 (71–93)	87 (70–131)	0.914
-1 h post-hemorrhage	111 (106–115)	95 (79–95)	0.057
-2 h post-resuscitation	91 (75–91)	89 (79–120)	0.857
Peak thrombin generation, nM:			
-Pre-treatment	66 (47–93)	101 (56–121)	0.286
-1 h post-hemorrhage	93 (61–137)	41 (19–56)	0.032
AUC thrombin generation, nM × min:			
-Pre-treatment	329 (252–329)	406 (254–406)	0.999
-1 h post-hemorrhage	495 (495–495)	294 (160–427)	0.4

^#^*p*-value by non-parametric Mann–Whitney test. All values are median (with interquartile range –IQR unless stated otherwise). CT-INTEM (*p* = 0.008), MCF-INTEM (*p* = 0.001), and MCF-EXTEM (*p* = 0.001) all significantly differed with time. AUC = area under curve; CT = clotting time; MCF = maximum clot firmness; PFA = platelet function analyzer.

**Table 4:** Arterial and venous blood gas parameters during the experiment (*n* = 12).

Variables	Resveratrol Treated (*n* = 6)	Control (*n* = 6)	*p*-Value ^#^
Arterial pH:			
-Pre-hemorrhage	7.44 (7.41–7.48)	7.46 (7.40–7.52)	0.696
-1 h post-hemorrhage	7.24 (7.21–7.33)	7.35 (7.33–7.36)	0.011
-1 h post-resuscitation	7.33 (7.32–7.38)	7.35 (7.31–7.39)	0.913
-2 h post-resuscitation	7.35 (7.34–7.39)	7.36 (7.32–7.42)	0.853
P_a_O_2_ (mmHg):			
-Pre-hemorrhage	187 (178–191)	196 (179–2.8)	0.329
-1 h post-hemorrhage	133 (124–153)	147 (130–170)	0.485
-1 h post-resuscitation	170 (146–207)	150 (132–163)	0.147
-2 h post-resuscitation	159 (152–174)	157 (138–172)	0.699
P_a_CO_2_ (mmHg):			
-Pre-hemorrhage	33.1 (28.4–38.5)	32.1 (26.7–36.0)	0.662
-1 h post-hemorrhage	45.9 (36.9–51.6)	37.9 (36.1–40.7)	0.162
-1 h post-resuscitation	43.2 (40.4–46.4)	44.7 (38.9–48.0)	>0.999
-2 h post-resuscitation	41.7 (37.0–43.7)	41.3 (37.0–46.2)	>0.999
Arterial HCO_3_ (mmol/L)			
-Pre-hemorrhage	22.1 (20.8–23.7)	22.3 (21.2–23.3)	0.892
-1 h post-hemorrhage	19.5 (17.1–20.9)	20.8 (18.3–22.3)	0.225
-1 h post-resuscitation	22.5 (21.2–23.6)	23.6 (22.9–24.2)	0.121
-2 h post-resuscitation	21.8 (20.9–23.4)	23.2 (22.7–23.9)	0.178
Arterial Base excess (mmol/L):			
-Pre-hemorrhage	−1.7 (−2.3–(−0.4))	−1.5 (−2.3–(−0.2))	0.792
-1 h post-hemorrhage	−6.9 (−8.3–(−5.0))	−4.0 (−6.7–(−2.9))	0.132
-1 h post-resuscitation	−2.4 (−3.7–(−1.6))	−1.5 (−2.2–(−0.5))	0.143
-2 h post-resuscitation	−2.9 (−3.6–(−1.5))	−1.7 (−2.3–(−0.7))	0.102
S_a_O_2_ (%):			
-Pre-hemorrhage			
-1 h post-hemorrhage	All values 100%, at all time points		
-1 h post-resuscitation			
-2 h post-resuscitation			
Central venous pH:			
-Pre-hemorrhage	7.41 (7.39–7.46)	7.43 (7.38–7.51)	0.792
-1 h post-hemorrhage	7.23 (7.16–7.28)	7.32 (7.29–7.32)	0.047
-1 h post-resuscitation	7.29 (7.28–7.34)	7.32 (7.30–7.36)	0.29
-2 h post-resuscitation	7.32 (7.30–7.35)	7.34 (7.29–7.39)	0.771
P_v_O_2_ (mmHg):			
-Pre-hemorrhage	46.1 (39.9–51.2)	46.5 (43.8–50.4)	0.931
-1 h post-hemorrhage	25.4 (19.2–29.3)	25.9 (23.8–27.7)	0.784
-1 h post-resuscitation	51.4 (45.1–59.6)	50.4 (46.4–53.0)	0.732
-2 h post-resuscitation	50.3 (44.9–59.0)	46.9 (41.9–49.9)	0.24
P_v_CO_2_ (mmHg):			
-Pre-hemorrhage	38.3 (33.8–42.1)	33.4 (29.3–40.0)	0.247
-1 h post-hemorrhage	59.3 (47.4–70.0)	49.1 (45.6–51.2)	0.18
-1 h post-resuscitation	50.4 (48.4–51.7)	48.5 (43.2–52.0)	0.589
-2 h post-resuscitation	47.1 (44.8–50.4)	48.0 (42.3–53.2)	0.181
Central venous HCO_3_ (mmol/L)			
-Pre-hemorrhage	24.0 (23.0–25.1)	23.4 (20.8 - 24.6)	0.699
-1 h post-hemorrhage	23.0 (21.7–25.0)	24.1 (21.4–25.4)	0.667
-1 h post-resuscitation	23.3 (22.9–25.0)	24.5 (23.3–24.9)	0.407
-2 h post-resuscitation	23.2 (22.6–24.5)	24.7 (23.1–25.2)	0.37
Venous Base excess (mmol/L):			
-Pre-hemorrhage	0.9 (−0.8–1.4)	−0.5 (−3.1–0.8)	0.306
-1 h post-hemorrhage	−3.8 (−5.5–(−1.2))	−1.6 (−4.8–(−0.1))	0.589
-1 h post-resuscitation	−2.5 (−2.8–(−0.5))	−1.3 (−1.8–(−0.4))	0.234
-2 h post-resuscitation	−2.2 (−2.6–(−0.1))	−1.1 (−1.7–(−0.5))	0.31
S_v_O_2_ (%):			
-Pre-hemorrhage	88.2 (76.3–90.8)	91.7 (85.2–92.6)	0.162
-1 h post-hemorrhage	29.3 (22.9–38.5)	41.0 (34.1–49.9)	0.064
-1 h post-resuscitation	84.0 (59.4–86.2)	85.1 (80.1–90.0)	0.589
-2 h post-resuscitation	84.2 (82.1–88.0)	79.6 (72.4–86.9)	0.589
Hemoglobin-venous (g/dL):			
-Pre-hemorrhage	13.5 (12.7–13.7)	15.8 (14.5–16.1)	0.262
-1 h post-hemorrhage	17.2 (14.9–19.5)	17.5 (15.8–18.1)	0.965
-1 h post-resuscitation	8.7 (7.1–10.7)	9.2 (9.0–9.5)	0.662
-2 h post-resuscitation	8.8 (7.3–10.8)	9.3 (8.8–9.5)	0.79
Sodium (mmol/L):			
-Pre-hemorrhage	145 (145–147)	146 (145–148)	>0.999
-1 h post-hemorrhage	144 (141–145)	145 (143–146)	0.298
-1 h post-resuscitation	144 (143–146)	145 (145–146)	0.141
-2 h post-resuscitation	144 (143–145)	145 (144–147)	0.175

^#^*p*-value by non-parametric Mann–Whitney test. All values are median (with interquartile range –IQR unless stated otherwise).

**Table 5:** Hemodynamic parameters, bleeding volume and intravenous fluid administered during the experiment (*n* = 12).

Variables	Resveratrol treated (*n* = 6)	Control (*n* = 6)	*p*-Value ^#^
Bleeding volume, mL	63.8 (57.8–78.6)	54.9 (42.5–58.9)	0.041
Total intravenous fluid volume, mL:			
- Compound Sodium Lactate	978 (924.8–1004.3)	925 (013.5-942)	0.31
- Colloid	652 (616.5-669.5)	617 (609-628)	0.31
Heart rate, beats/min:			
-Pre-hemorrhage	86 (52–122)	104 (93–118)	0.485
-1 h post-hemorrhage	204 (179–221)	182 (156–208)	0.31
-1 h post-resuscitation	172 (153–184)	150 (134–171)	0.18
-2 h post-resuscitation	176 (156–189)	154 (126–167)	0.093
Mean arterial pressure, mmHg:			
-Pre-hemorrhage	60 (59–68)	65 (60–74)	0.31
-1 h post-hemorrhage	33 (31–37)	39 (38–40)	0.041
-1 h post-resuscitation	76 (71–81)	76 (68–79)	0.589
-2 h post-resuscitation	71 (67–76)	69 (64–73)	0.394
Cardiac index, mL/min/m^2^:			
-Pre-hemorrhage	95 (90–104)	86 (74–93)	0.082
-1 h post-hemorrhage	36 (32–40)	41 (33–49)	0.18
-1 h post-resuscitation	118 (111–130)	134 (120–170)	0.093
-2 h post-resuscitation	121 (115–139)	143 (119–152)	0.31
Oxygen extraction ratio, %:			
-Pre-hemorrhage	13 (12–23)	11 (10–17)	0.24
-1 h post-hemorrhage	71 (62–77)	60 (50–66)	0.065
-1 h post-resuscitation	19 (16–20)	17 (12–22)	0.699
-2 h post-resuscitation	18 (14–20)	22 (15–29)	0.589

^#^ *p*-value by non-parametric Mann–Whitney test. All values are median (with interquartile range –IQR unless stated otherwise).

**Table 6:** Linear mixed models showing the associations between resveratrol treatment with maximum clot firmness on the Rotational Thromboelastometry (ROTEM^®^) of the anesthetized greyhound dogs before and after induced hemorrhage (*n* = 12) after adjusting for volume of blood removed using unstructured covariance structure. MCF = maximum clot firmness.

Predictors	Coefficient (95% Confidence Interval)	*p*-Value
MCF-InTEM		
Resveratrol treatment	10.6 (1.0–20.3)	0.034
Volume of blood removed	0.1 (0.3 to −0.3)	0.673
Time points	−4.7 (−3.3 to −6.1)	0.001
Resveratrol × time	1.8 (−0.3 to 3.7)	0.078
MCF-ExTEM		
Resveratrol treatment	5.5 (−2.8 to 13.8)	0.158
Volume of blood removed	−0.4 (−0.1 to −0.7)	0.028
Time points	−0.8 (−2.3 to 0.7)	0.260
Resveratrol × time	2.9 (0.7 to 5.1)	0.017

## Data Availability

The data presented in this study are available on request from the corresponding author.
